# NF-κB Signalling in Glioblastoma

**DOI:** 10.3390/biomedicines5020029

**Published:** 2017-06-09

**Authors:** Vincent Soubannier, Stefano Stifani

**Affiliations:** Department of Neurology and Neurosurgery, Montreal Neurological Institute, McGill University, Montreal, QC H3A2B4, Canada; vincent.soubannier@mcgill.ca

**Keywords:** brain cancer, cancer stem-like cells, DNA damage repair, glioblastoma, mesenchymal glioblastoma subtype, NF-κB (nuclear factor-κB)

## Abstract

Nuclear factor-κB (NF-κB) is a transcription factor regulating a wide array of genes mediating numerous cellular processes such as proliferation, differentiation, motility and survival, to name a few. Aberrant activation of NF-κB is a frequent event in numerous cancers, including glioblastoma, the most common and lethal form of brain tumours of glial cell origin (collectively termed gliomas). Glioblastoma is characterized by high cellular heterogeneity, resistance to therapy and almost inevitable recurrence after surgery and treatment. NF-κB is aberrantly activated in response to a variety of stimuli in glioblastoma, where its activity has been implicated in processes ranging from maintenance of cancer stem-like cells, stimulation of cancer cell invasion, promotion of mesenchymal identity, and resistance to radiotherapy. This review examines the mechanisms of NF-κB activation in glioblastoma, the involvement of NF-κB in several mechanisms underlying glioblastoma propagation, and discusses some of the important questions of future research into the roles of NF-κB in glioblastoma.

## 1. Glioblastoma

Glioblastoma (GBM), the most common and malignant of all gliomas, is a brain cancer characterized by remarkable cellular heterogeneity, aggressive growth, extensive invasion of brain tissue, and almost inevitable recurrence. It is virtually untreatable, even after combined surgery, radiation therapy and chemotherapy, and GBM patients have an average survival of less than two years [1,2,3].

GBM exhibits a build-up of a variety of poorly differentiated neural cells. At a minimum, GBM is hypothesized to contain three cell subpopulations. One pool is thought to include relatively rare cells with self-renewing and cancer (re)populating potential (hereafter referred to as “stem-like cells”). A separate pool, also expected to be infrequent, is proposed to harbour cells that have exited the cell cycle, have slow cellular turnover, but are not endowed with persistent self-renewal capacity (hereafter referred to as “quiescent cells”). The third pool is thought to be more prevalent and diverse and to include a collection of mitotic cells exhibiting various degrees of incomplete differentiation (hereafter operationally defined collectively as “differentiated cells”). The GBM stem-like cell pool is thought to be responsible for tumour (re)initiation. Both stem-like and quiescent cell pools have the potential to act as reservoirs of undifferentiated cells able to give rise to more rapidly dividing progeny cells. The differentiated GBM cell pool is considered as the main contributor to processes underlying GBM development, such as tumour growth and invasion [4,5,6,7,8,9].

The cellular heterogeneity of GBM is a major obstacle on the road to treatment because the diversity of cancer cells within the same tumour implies that they may respond differently to therapy. Current GBM treatments usually target the differentiated cell pools. Unfortunately, these approaches have failed to treat GBM. This situation suggests that stem-like and quiescent cell pools are not targeted by current therapies and may represent the population(s) responsible for GBM therapy resistance and recurrence after surgery and treatment. Unfortunately, our understanding of the pathobiology, and contribution to tumourigenesis, of different GBM cell pools remains limited.

To add to the complexity of GBM, emerging evidence suggests that at least some of the different GBM cell populations are in a fluent state and can switch from one phenotype to another. In this regard, recent studies suggest that exposure of glioma cells to therapeutic doses of temozolomide, the compound most commonly used in GBM chemotherapy, increases the stem-like cell pool as a result of a “differentiated-to-stem-like” phenotypic shift [9,10,11]. These observations suggest the existence of dynamic relationships between different GBM cell pools, a situation presenting a remarkable challenge to current and future GBM therapies. These findings underscore the necessity to improve our understanding of the molecular mechanisms underlying the pathobiology of different GBM cell populations and their contributions to gliomagenesis.

Numerous oncogenic pathways are active in GBM, and several previous reviews have addressed how they contribute to gliomagenesis by promoting processes ranging from proliferation to invasion to therapy resistance, e.g., [2,8,9,12,13]. This review will focus specifically on the involvement of nuclear factor-κB (NF-κB) signaling pathways in GBM and the implications of aberrant NF-κB activation in different GBM cell populations.

## 2. Nuclear Factor-κB Signalling

The term NF-κB signalling refers to several mechanisms, activated by a variety of stimuli, which ultimately converge on the NF-κB family of transcription factors. NF-κB is a dimeric DNA-binding complex composed of varying combinations of five family members including p50/NFKB1 (p50 hereafter for sake of clarity), p52/NFKB2 (p52), Rel-like domain containing protein A (RelA/RELA (RelA), RelB/RELB (RelB), and c-Rel/REL (c-Rel) [14,15,16,17,18,19]. These molecules act frequently as heterodimers (e.g., p50:RelA), but homodimers (e.g., RelA:RelA) have also been observed. They regulate the expression of a wide array of genes involved in important biological processes, such as cell proliferation, apoptosis, DNA repair, and immune and inflammatory responses, to name a few [14,15,16,17,18,19].

NF-κB dimers are expressed in many different cell types, where they are usually kept in an inactive (non-DNA bound) state by specific inhibitors of NF-κB (IκB) until the reception of activating signals occurs. IκB proteins such as NF-κB Inhibitor Alpha (NFKBIA)/IκBα (IκBα) bind NF-κB dimers and sequester them in the cytosol, thereby preventing DNA binding and transcriptional regulation by the latter.

In canonical NF-κB pathways, the activation of the IκB kinase (IKK/IKBK) complex (IKK) [composed of IKKα, IKKβ, and NF-κB essential modulator (NEMO)/IKKγ subunits] leads to IκBα phosphorylation by IKKβ, ubiquitination and proteosomal degradation of IκBα, and the ensuing dissociation of p50:RelA-containing NF-κB dimers from IκB and the translocation of NF-κB to the nucleus [14,15,16,17,18,19]. Canonical NF-κB activation is typically observed after stimulation of surface receptors such as the tumor necrosis factor alpha (TNFα) receptor 1 or the interleukin-1 receptor [14,15,16,17,18,19].

Activation of NF-κB can also occur through non-canonical mechanisms, involving NF-κB-inducing kinase (NIK; referred to as Mitogen-Activated Protein Kinase Kinase Kinase 14 (MAP3K14) in human cells) and p52-containing NF-κB dimers. In these pathways, acting downstream of receptors like the B cell activating factor receptor or the lymphotoxin beta receptor, phosphorylation of the p52 precursor form, p100 (which retains p100:RelB-containing NF-κB in the cytosol), leads to p100 proteolytic cleavage, generating p52:RelB NF-κB dimers that can translocate to the nucleus [14,15,16,17,18,19].

A third mode of NF-κB activation, termed atypical, has also been described, most characteristically in response to DNA double-strand breaks, replication stress or reactive oxygen species. Atypical NF-κB activation mechanisms can be divided into two main groups, depending on whether they are dependent or independent of the activity of the IKK complex [14,20]. In the case of IKK-dependent atypical pathways, stressful stimuli like genotoxic stress induce the NEMO protein to translocate to the nucleus, where it becomes sumoylated and ubiquitinated through a mechanism dependent on the ataxia and telangiectasia mutated (ATM) kinase. The sequential phosphorylation and ubiquitination of sumoylated nuclear NEMO results in the export of NEMO from the nucleus to the cytosol, where the latter activates IKKβ and induces NF-κB. IKK-independent atypical pathways involve p50-containing dimers but rely on other kinases, such as casein kinase-II, for the dissociation of NF-κB dimers from IκB and translocation to the nucleus [14,20].

Regulation of NF-κB signalling does not only involve nuclear translocation but also includes several other control mechanisms, ranging from post-translational modifications of specific NF-κB subunits, protein-protein interactions occurring at specific gene regulatory sites, as well as mechanisms of nuclear export [14,15,16,17,18,19,20]. Thus, different cellular responses to NF-κB usually result from a complex combination of specific mechanisms that may differ in their upstream stimuli and/or downstream targets.

## 3. Activation of NF-κB in Glioblastoma

Activation of NF-κB is a frequent event in several tumours. Deregulated NF-κB activation is often oncogenic through the promotion of tumor growth and invasion, the suppression of programmed cell death, as well as resistance to therapy [21,22]. Consistent with the complex nature of NF-κB activation in response to a variety of stimuli, aberrant NF-κB activity in cancer may ensue as a result of numerous events, including mutation or deregulated expression of genes encoding the NF-κB proteins or, more frequently, the perturbation of the mechanisms controlling the activation of NF-κB dimers [21,22,23,24,25].

Aberrant constitutive activation of NF-κB is a common event in GBM [26,27,28,29,30]. Numerous mechanisms have been proposed to contribute to deregulated NF-κB signalling in gliomas. For instance, receptor tyrosine kinases, most notably epidermal growth factor receptor (EGFR) and platelet derived growth factor receptor (PDGFR), which are often aberrantly activated in GBM, have been linked to NF-κB activation through a number of mechanisms, involving both protein kinase B/AKT (AKT)-dependent and -independent pathways. Oncogenic EGFR and PDGFR signalling mechanisms are important contributors to tumour growth and invasion in GBM, and NF-κB is implicated in at least some of the tumour promoting functions of these receptors [31,32,33,34,35].

Loss of tumour suppressors such as phosphatase and tensin homolog (PTEN) and neurofibromin 1 (NF1) has also been linked to aberrant NF-κB activation in GBM, at least in part as a result of increased PI3-kinase (PI3K) activity [32,35]. The depletion of the tumour suppressor, Krueppel-like factor 6 (KLF6), which acts as a negative NF-κB regulator, is also postulated to contribute to NF-κB activation in GBM [36], and so are perturbations of *TP53* biology, including both the loss and activation of the *TP53*-encoded protein, p53 [37,38,39,40].

Numerous other mechanisms are suggested to contribute to aberrant NF-κB signalling in GBM, including NF-κB activation by peptidyl-prolyl isomerase PIN1, mixed lineage kinase 4 (MLK4), heterozygous deletion of *NFKBIA*, the gene encoding IκBα, high levels of micro(mi)RNA-30e*, which targets IκBα, as well as DNA-damage [41,42,43,44,45].

These combined observations underscore the involvement of aberrant NF-κB pathways in multiple processes of GBM pathogenesis and identify several mechanisms upstream and downstream of NF-κB signalling.

## 4. Role of NF-κB in Glioblastoma Stem-Like Cells

### 4.1. Glioblastoma Patient-Derived Stem-Like Cell Cultures

Recent advances in GBM research have enabled the derivation of cultures of stem-like cells from surgically resected brain tumours from individual patients. These patient-derived cells exhibit stem-like cell behaviour in vitro and can serially propagate brain tumours when implanted into the crania of host mice under limiting dilution conditions [6,7,8,9]. GBM patient-derived stem-like cells (GSCs) share with normal neural stem cells the ability to generate progeny cultures comprised of a mixture of stem-like and more restricted non-stem-like descendants. This thereby offers the previously unavailable opportunity to study the behaviour of different GBM cell populations in mixed cultures initiated by single stem-like cells derived from individual patients. This situation provides a physiologically informative experimental model system to study mechanisms underlying the behaviour of different GBM cell pools contributing to tumour initiation, development and recurrence.

### 4.2. Involvement of NF-κB in Glioblastoma Stem-Like Cell Maintenance

Although the characterization of the precise pattern of NF-κB activation in different GBM cell populations remains elusive in surgically resected tissues, several studies have demonstrated the activation of NF-κB in patient-derived GSC cultures [42,46,47,48,49]. The involvement of NF-κB in GSCs has been examined using a number of biological assays, including studies based on the ability of cancer cells with stem-like properties to give rise to new populations of descendant cells (referred to as “tumourspheres”) under limiting dilution conditions. In these assays, the number of cells/well required to generate at least one new tumoursphere is taken as an indication of the frequency of cells with repopulating ability, a typical feature of stem-like cells.

The inhibition of endogenous NF-κB activity in patient-derived GSC cultures, using the selective IKKβ antagonist, Compound A, or siRNA-mediated knockdown of IKKβ and/or RelA, was shown to markedly decrease tumoursphere formation frequency, suggesting that canonical NF-κB inhibition causes a reduction in the number of cells with self-renewal capacity. A similar effect was observed when the non-canonical NF-κB pathway was impaired through the attenuation of p52 or RelB [48]. A role for non-canonical NF-κB signalling in GSC self-renewal was also suggested by the independent observation that knockdown of RelB blocks the self-renewal activity of patient-derived glioma-initiating cells [49]. Together, these studies suggest that NF-κB signalling is involved in sustaining the GBM stem-like cell compartment and that both canonical and non-canonical branches of the NF-κB pathway are important for this function.

Rinkenbaugh and colleagues suggested that at least one source of NF-κB activation in GSCs is the transforming growth factor-β-activated kinase 1, a protein previously implicated in NF-κB signalling due to its ability to activate IKK [48]. Ohtsu et al. provided evidence suggesting that non-canonical NF-κB signalling is activated in GSCs downstream of epithelial V-like antigen 1, a protein originally identified as an immunoglobulin superfamily member expressed in developing thymus epithelial cells and involved in T-cell development in early mouse embryos. Epithelial V-like antigen 1 activates the non-canonical NF-κB signaling pathway through a TNF receptor-associated factor 2/cellular inhibitor of apoptosis-dependent accumulation of NIK [49].

Taken together, these observations suggest that a variety of mechanisms can lead to aberrant NF-κB activation in GSCs, consistent with the above-mentioned demonstration that a plethora of deregulated processes, including EGFR amplification, PTEN deletion, and monoallelic *NFKBIA* deletion, have been associated with deregulated NF-κB activation in GBM. It is therefore plausible that a variety of NF-κB-regulated mechanisms, activated in response to both canonical and non-canonical pathways, contribute to the regulation of the behaviour of GSCs.

The pathophysiological significance of these observations remains to be fully determined. NF-κB inhibition might result in depletion of the GBM stem-like cell pool because of impaired survival of these cells. Alternatively, the inhibition of NF-κB signalling in GSC cultures might enhance the pool of senescent cells at the expense of the stem-like compartment, a possibility that would be consistent with the demonstration that blockade of NF-κB signalling drives differentiating glioblastoma cells into replicative senescence [50]. It seems less likely that the stem-like cell compartment might be diminished as a result of an enhanced transition to a more proliferative, “transit-amplifying-like”, state because the growth rate of NF-κB-attenuated GSCs was shown to be decreased, rather than increased, in ex vivo brain slice explants [48]. In the future, it will be important to characterize further the mechanisms upstream and downstream of NF-κB activation in the GSC compartment, as well as to perform in vivo brain tumour xenograft studies under limiting dilution conditions to better understand the contribution of NF-κB to stem-like cells and to GBM initiation and/or recurrence. 

## 5. Involvement of NF-κB in Glioblastoma Invasion

### 5.1. Deregulated NF-κB Activation in Mesenchymal GBM Subtype

It has long been established that epithelial to mesenchymal transition (EMT) is a process associated with advanced malignancy in numerous cancers, and that NF-κB acts to promote EMT in several cell types [51]. Consistent with the latter finding, the examination of glioma patient-based mRNA expression databases first suggested that aberrant NF-κB activation was preferentially associated with GBM cases with a mesenchymal phenotype, rather than GBM subtypes with more neural features (many of which are defined as having a proneural phenotype) [52,53,54]. Mesenchymal features are a hallmark of glioma aggressiveness and are associated with poor patient outcome, due in part to the highly invasive nature of these tumours and increased radioresistance [55].

An EMT-like proneural-to-mesenchymal phenotypic shift occurs in GBM in response to factors in the microenvironment or cytotoxic treatments. This transition involves NF-κB, as demonstrated by the observation that canonical TNFα/NF-κB signalling can promote a proneural-to-mesenchymal transition in at least a subset of GBM patient-derived GSC cultures [42,47]. Moreover, RelB is highly expressed in the mesenchymal glioma subtype and the loss of RelB significantly attenuates glioma cell survival, motility and invasion. Importantly, RelB promotes the expression of mesenchymal genes in glioma cells [56]. Consistent with these findings, a mesenchymal signature, including NF-κB activation, is correlated with poor radiation response and shorter survival in GBM patients [47,56].

Further evidence for an important role for NF-κB in mesenchymal identity in GBM was provided by the demonstration that RelB-mediated NF-κB signalling is a critical mediator of GBM cell migration and invasion stimulated by the SMAC (second mitochondrial activator of caspases) mimetic, BV6, a molecule that antagonizes the inhibitor of apoptosis proteins and also triggers cell elongation, migration and invasion in GBM [57]. BV6-stimulated NF-κB activation leads to elevated *TNFα* mRNA levels, as well as increased levels of NF-κB target genes implicated in cell migration and invasion [57].

The mechanisms responsible for the activation of NF-κB in mesenchymal GBM tumours are beginning to be elucidated. The kinase MLK4 is overexpressed in mesenchymal, but not proneural, GSCs, where it is important for maintenance of the mesenchymal phenotype and for self-renewal, motility, tumourigenesis, and radioresistance. MLK4 binds to, and phosphorylates, IKKα in GSC cultures, thereby leading to activation of NF-κB signalling [42]. The peptidyl-prolyl isomerase PIN1, which recognizes phosphorylated Ser residues on RelA and promotes NF-κB activation, is up-regulated in GBM [41]. PIN1 attenuation decreases the amount of activated, phosphorylated RelA in the nucleus, with a concomitant decrease in the expression of the NF-κB target gene, *interleukin-8* (*IL-8*). These effects are associated with decreased glioma cell dissemination capacity. Conversely, two negative regulators of NF-κB signalling, inhibitor of growth family member 4 and protein inhibitor of activated STAT3, are decreased in GBM when compared to non-cancerous cells [58,59,60].

### 5.2. Involvement of NF-κB in Transcriptional Activation of Genes Promoting Epithelial-to-Mesenchymal Transition and Cell Motility 

As discussed above, NF-κB signalling pathways play key roles in promoting and maintaining EMT in both healthy and cancer cells. This function is performed through regulation of the expression of numerous epithelial and mesenchymal genes [51,61]. For instance, studies in both invertebrate and vertebrate species identified NF-κB as a key player in the transactivation of mesenchymal genes such as *Snail*, *zinc finger E-Box binding homeobox 1* (*ZEB1*), *ZEB2*, *Twist*, and *matrix metalloproteinase* (*MMP*)*-2* and *MMP-9* [51,60,61].

There is evidence suggesting that NF-κB also activates the expression of mesenchymal genes in GBM. NF-κB binds to the *ZEB1* promoter in glioma cells in response to connective tissue growth factor, which is important for glioma invasion [62]. NF-κB is also involved in the activation of *MMP-2* and *MMP-9* in GBM cells, at least in part in response to protein kinase C (PKC) and mechanistic target of rapamycin (mTOR) signalling [63,64]. Moreover, RelB promotes the expression of the gene *YKL-40*, considered as a typical marker of the mesenchymal GBM subtype [56].

NF-κB has been further implicated in promoting GBM invasion by the demonstration of its involvement in the transactivation of the expression of several genes encoding molecules promoting cell motility and invasion. These include *fibroblast growth factor inducible 14* (*FN14*), a member of the TNF receptor superfamily. *FN14* is highly expressed in invading glioma cells in vivo. The *FN14* promoter region contains NF-κB binding sites important for sustained overexpression of *FN14* and enduring glioma cell invasion. Consistently, *FN14* gene expression levels increase with glioma grade and inversely correlate with patient survival [65]. Activated NF-κB in GBM also regulates other genes involved in cell migration, such as *IL-8*, *monocyte chemoattractant protein 1*, *cxc chemokine receptor 4*, to name a few [57].

In addition to controlling gene regulatory events promoting glioma invasion in a cell-autonomous manner, NF-κB also appears to promote GBM cell invasion in a non-cell autonomous ways. Specifically, high endogenous expression of receptor activator of NF-κB (RANKL) in GBM cells leads to the activation of neighbouring astrocytes in the tumour microenvironment through NF-κB signalling. Activated astrocytes in turn signal back to the glioma cells to promote glioma invasion [66]. These findings are consistent with the demonstration that intercellular communication between neighbouring astrocytes and GBM cells, possibly mediated by secreted extracellular vesicles, plays key roles in GBM growth and invasion [67,68].

These combined observations provide evidence for an important role of NF-κB in the promotion of more invasive and malignant mesenchymal features in GBM. The interpretation of these observations is complicated in part by the lack of information about the pattern of NF-κB activation in different GBM cell subpopulations. Presumably, enhanced dissemination potential should be a feature of more developmentally advanced GBM cells resembling the migratory neural cells in the healthy brain (e.g., glial precursor cells that leave their place of origin in the subventricular zone to reach their final destinations in the brain). If this were indeed the case, it would be reasonable to assume that NF-κB signalling is activated in, and promotes the migration of, more differentiated GBM cellular subtypes. A similar scenario could also result, however, by the activation of NF-κB in the GBM stem-like cell compartment, where its ability to promote proneural-to-mesenchymal transition could result in the generation of more restricted daughter cells with enhanced dissemination potential. It is also possible that NF-κB could enhance the migratory potential of most GBM cells in which it is activated, regardless of their stem-like or non-stem-like state. In the future, it will be important to better characterize the features of the cancer cells in which NF-κB is activated in GBM surgical specimens, as well as in the heterogeneous population of cells comprising typical GBM tumourspheres studied in vitro.

## 6. Other Roles of NF-κB in Glioblastoma

### 6.1. Resistance to Radiotherapy

Radiotherapy is a customary component of GBM treatment. Unfortunately, the majority of GBM patients exhibit radioresistance. Several mechanisms of cancer cell resistance to radiotherapy have been described, including enhanced ability to repair DNA damage, the presence of quiescent or slowly dividing cells that are less vulnerable to DNA damage, and resistance to apoptosis [69,70]. Numerous lines of studies suggest that aberrant NF-κB activation may contribute to radioresistance in GBM by modulating several of these processes.

In response to DNA damage, cells usually activate mechanisms aimed at restoring genomic stability or, in the case of more severe damage, leading to apoptosis. DNA damage is a well-known activator of NF-κB signalling; in turn, NF-κB plays important roles in DNA damage repair mechanisms. For instance, the DNA damage sensor poly-(ADP-ribose) polymerase-1 (PARP1) activates NF-κB through an ATM- and IKK-mediated pathway. NF-κB also participates in DNA repair through interaction with breast cancer-associated gene 1 (BRCA1), at least in part via interaction with BRCA1-CtIP complexes, thereby promoting homologous recombination [44,71,72,73]. NF-κB activation also induces the expression of BRCA2, another important DNA repair proteins, as well as ATM, the key DNA double-strand-signalling kinase. Moreover, NF-κB activates the non-homologous end-joining recombination protein, ku70 [74,75]. It is worth mentioning that NF-κB also mediates chemoresistance to alkylating agents such as temozolomide. The mutagenic effects of these compounds are inhibited by the cellular DNA repair enzyme *O*-6-methylguanine-DNA-methyltransferase (MGMT), which removes alkyl/methyl adducts from DNA. Since MGMT becomes inactivated in the process, its de novo expression is a central mode of chemoresistance. NF-κB plays an important role in regulation of MGMT activity in glioma cells by activating *MGMT* gene expression through two NF-κB binding sites within the *MGMT* promoter [76].

As discussed above, NF-κB activation promotes maintenance of the GBM stem-like cell pool [48,49]. GBM stem-like cells are thought to promote radioresistance [77,78,79], suggesting that an additional mechanism through which NF-κB has an impact on GBM resistance to radiotherapy is by increasing the fraction of cancer cells with stem-like behaviour. The important role of NF-κB in promoting a mesenchymal GBM phenotype is also considered to be a contributor to radiation resistance because the mesenchymal subtype is associated with poor radiation response in GBM patients [47]. It is hypothesized that mesenchymal differentiation driven by NF-κB is associated with activation of checkpoint pathways, leading to enhanced DNA damage repair and unperturbed cell cycle progression in response to radiation [47].

Several lines of evidence suggest additional roles for NF-κB signalling in radiotherapy resistance in cancers by promoting the expression of genes implicated in evasion of apoptosis, the promotion of cell cycle progression, and the production of antioxidants [80,81]. NF-κB signalling regulates the expression of genes important for cell survival, including *Bcl2*, *Bcl-xL*, *survivin* and *inhibitor of apoptosis proteins*, as well as cell cycle progression genes like *Cyclin D1* [80,81,82,83]. Moreover, radiation-activated NF-κB signalling is associated with upregulated expression of NF-κB targets genes such as *IL-6* and *IL-8* in GBM. It was reported that IL-6 secreted by glioma cells enhances the invasive potential of these cells [84] and that IL-8 is important for glial tumour neovascularity and progression [85].

Together, these observations provide evidence for a role of NF-κB in numerous mechanisms underlying resistance to radiation therapy in several cancers, including GBM. These processes are likely to be particularly relevant in the context of the GBM stem-like cell pool, but may also impact on other GBM subpopulations in which NF-κB is activated.

### 6.2. Regulation of Cell Metabolism

As is the case in other cancers, aerobic glycolysis (production of lactate from glucose in the presence of oxygen) is a hallmark of GBM, underlying the need to synthesize molecules essential for tumour cell proliferation, including nucleotides, fatty acids, and proteins [86]. Consistent with the observation that NF-κB signalling plays an important role in regulating energy metabolism in several cancers [87,88], NF-κB contributes to the promotion of aerobic glycolysis in GBM through a number of mechanisms.

Pyruvate kinase M2 (PKM2), a member of the enzyme family regulating the rate-limiting step of glycolysis, is overexpressed in numerous cancers, including GBM [89,90]. Activation of EGFR in cancer cells results in increased glucose uptake and lactate production in a PKM2-dependent manner. The aberrant activation of *PKM2* expression occurs in response to EGFR-induced and PKCε monoubiquitylation-dependent activation of RelA, which interacts with transcription factor hypoxia-inducible factor 1-α to form a dimer that can bind to the *PKM2* promoter and activate *PKM2* transcription. Consistent with these findings, *PKM2* expression correlates with EGFR and IKKβ activity in human GBM specimens and with the grade of glioma malignancy [34].

NF-κB could also contribute to energy metabolism in GBM by upregulating mitochondrial respiration. In the presence of functional p53, RelA activates the expression of mitochondrial SCO2 (synthesis of cytochrome c oxidase assembly protein), a metallochaperone essential in the biogenesis of cytochrome c oxidase subunit II, enhancing oxidative metabolism [91]. Although this process may be altered in cancer cells without a functional p53, resulting in lower oxidative phosphorylation and increased glycolysis (possibly due to upregulation of metabolic genes such as high affinity glucose transporter 3), the possible significance of these mechanisms in the context of GBM remains to be defined.

In addition to glycolysis, glutamine metabolism is also important in cancer cells, both as another major energy source through α-ketoglutarate and as a nitrogen donor for nucleic acid synthesis. Several lines of evidence suggest an involvement of NF-κB in the control of glutamine metabolism. Glutaminase is likely the rate-limiting enzyme for glutamine consumption in cancer cells and increased glutaminase activity by Rho GTPase through a NF-κB-dependent mechanism was described as a process involved in meeting the elevated glutamine demand in cancer cells [92]. Additionally, NF-κB may exert a control on the expression of glutaminase in cancer cells through the regulation of miRNAs. In human leukemic Jurkat cells, RelA binds to the *miR-23a* promoter and inhibits *miR-23a* expression. In turn, *miR-23* targets *glutaminase* mRNA and inhibits expression of *glutaminase* [93].

Taken together, these findings point to NF-κB as an emerging important regulator of cell metabolism in numerous cancers, including GBM.

### 6.3. Involvement in Autophagy

Autophagy is an important cellular process that generally protects cells from stressful conditions by enabling continuous overhaul of cellular constituents through the degradation and recycling of damaged or non-essential macromolecular components, or even entire organelles such as mitochondria, ribosomes or endoplasmic reticulum [94,95,96]. Autophagy can play tumour-suppressing roles by maintaining cellular homeostasis through protection from accumulation of damaged proteins or reactive oxygen species. In cancer cells, however, autophagy plays a variety of tumour-promoting roles, including supporting cellular metabolism to promote cancer cell growth and survival and contributing to therapy resistance. Autophagy can also supports metastasis by protecting detached cells from anoikis, a form of programmed cell death [96,97,98,99,100].

The enhancement of pro-survival autophagy mechanisms is considered as an important contributor to cancer-promoting metabolism alterations in GBM. The remarkable resistance of GBM to chemo- and radiotherapy is also thought to result in part from the contribution of autophagy to the adaptation capabilities of this cancer [99,100,101]. On the basis of these considerations, the goal of inhibiting mechanisms of autophagy is emerging as an attractive therapeutic approach in GBM.

Recent studies suggest a complex interplay between NF-κB and autophagy [102]. NF-κB was initially proposed to have a negative effect on autophagy by activating the autophagy inhibitor mTOR [103,104]. However, more recent work has provided evidence that autophagy can activate NF-κB [105]. In agreement with this finding, there is evidence that autophagy and NF-κB mechanisms may cooperate in gliomas. Specifically, the mediator of autophagy, multifunctional scaffold protein p62, plays a role in the activation of IKKs and the resulting activation of NF-κB signaling [106,107,108]. A complex cross-talk between NF-κB and autophagy is further suggested by the observation that selenite-induced decrease in the expression of heat shock protein-90, which is associated with decreased autophagy and increased apoptosis, inhibits NF-κB signaling in human leukemia cells [109]. Consistently, selective targeting of heat shock protein-90 resulting in compensatory autophagy and unfolded protein response in mitochondria is correlated with the repression of NF-κB-dependent gene expression, enhanced tumor cell apoptosis, and reduced intracranial GBM growth in mice [110]. These observations suggest an important involvement of NF-κB in regulating the balance between autophagy and apoptosis in cancer.

In conclusion, these findings underscore important, but complex, interactions between autophagy and NF-κB during key cellular processes in cancer, including regulation of cellular metabolism, resistance to chemo- and radiotherapy, and/or responses to unfolded proteins. Understanding these mechanisms may enable more effective combinatorial therapeutic strategies.

### 6.4. Promotion of Angiogenesis

Angiogenesis is a common feature of the most aggressive gliomas and is correlated with poor patient prognosis. As discussed above, NF-κB activates the expression of *IL-8*, a pro-angiogenic gene, in glioma cells [85]. Moreover, NF-κB promotes the expression of vascular endothelial growth factor (VEGF), a major driver of angiogenesis [111]. Consistently, the impairment of NF-κB signaling significantly decreases GBM growth and angiogenesis in nude mice [111]. During the process of glioma angiogenesis, NF-κB is induced downstream of the transcription factor, BMI1 proto-oncogene, polycomb ring finger (BMI1), whose activity is required for expression of both NF-κB and VEGF [112]. Both the inhibition of NF-κB activity and knockdown of BMI1 result in decreased angiogenesis in orthotopically transplanted human gliomas [112]. These findings are in agreement with the demonstrated involvement of NF-κB in angiogenesis in other cancers [113] and further underscore the multiple roles of this transcription factor in gliomas.

Lastly, it is worth noting that several studies have shown that GSCs have the capacity to trans-differentiate into endothelial cells (ECs) that exhibit EC molecular profiles in vitro and can contribute to GBM vascularization in vivo [114,115,116,117]. It is possible that NF-κB may be involved in the EC trans-differentiation potential of GSCs. This is suggested by the recent demonstration that ovarian cancer stem-like cells can be induced to trans-differentiated into ECs by the activation of NF-κB through an autocrine loop mediated by the chemokine CCL5 [118]. CCL5 is synthesized and secreted by glioma-associated microglia [119], and thus a CCL5-mediated activation of NF-κB in GSCs may contribute to EC trans-differentiation in GBM, as is the case in other cells.

## 7. Concluding Comments

Aberrant NF-κB activation is a hallmark of numerous cancers, including GBM. This situation is consistent with the involvement of NF-κB-mediated signalling pathways in the control of a broad range of biological processes, including cell proliferation, survival, differentiation, motility, DNA repair, inflammation and angiogenesis. The fact that multiple stimuli and conditions can lead to NF-κB activation in GBM, often triggering multiple NF-κB pathways concurrently, implies that different modes of NF-κB activation may impact specifically on different mechanisms of cancer propagation. Thus, it will be of particular importance in the future to precisely understand not only the upstream stimuli but also the downstream targets of NF-κB signalling pathways during the various processes underlying gliomagenesis.

This information will also be essential if NF-κB-mediated pathways are to be considered as potentially attractive targets for GBM therapy. This possibility has been the subject of substantial interest, as addressed extensively in several previous reviews [21,23,30,120,121]. It should be emphasized, however, that although a number of approaches targeting NF-κB have shown promise in preclinical GBM models, those strategies that have been tested in clinical settings have thus far not shown satisfactory advantages over other approaches (reviewed in [30,120] and references therein). The promises and challenges of intervention strategies targeting NF-κB pathways will rely on the ability to design approaches directed at specific physiological mechanisms responsible for specific pathological outcomes in response to NF-κB activation. Moreover, given the role played by NF-κB in several vital processes, such as cellular homeostasis and immunity, prolonged NF-κB inhibition may possibly result in detrimental effects in tissues other than the cancerous ones.

In summary, understanding the range of contributions of NF-κB pathways to GBM both represents a remarkable scientific challenge and has the potential to provide important new insight into the management of this deadly cancer.
